# Functional Amyloid Formation within Mammalian Tissue

**DOI:** 10.1371/journal.pbio.0040006

**Published:** 2005-11-29

**Authors:** Douglas M Fowler, Atanas V Koulov, Christelle Alory-Jost, Michael S Marks, William E Balch, Jeffery W Kelly

**Affiliations:** **1** Department of Chemistry and The Skaggs Institute of Chemical Biology, The Scripps Research Institute, La Jolla, California, United States of America; **2** Department of Cell Biology and the Institute for Childhood and Neglected Diseases, The Scripps Research Institute, La Jolla, California, United States of America; **3** Department of Pathology and Laboratory Medicine, University of Pennsylvania School of Medicine, Philadelphia, Pennsylvania, United States of America; University of California, San FranciscoUnited States of America

## Abstract

Amyloid is a generally insoluble, fibrous cross-β sheet protein aggregate. The process of amyloidogenesis is associated with a variety of neurodegenerative diseases including Alzheimer, Parkinson, and Huntington disease. We report the discovery of an unprecedented functional mammalian amyloid structure generated by the protein Pmel17. This discovery demonstrates that amyloid is a fundamental nonpathological protein fold utilized by organisms from bacteria to humans. We have found that Pmel17 amyloid templates and accelerates the covalent polymerization of reactive small molecules into melanin—a critically important biopolymer that protects against a broad range of cytotoxic insults including UV and oxidative damage. Pmel17 amyloid also appears to play a role in mitigating the toxicity associated with melanin formation by sequestering and minimizing diffusion of highly reactive, toxic melanin precursors out of the melanosome. Intracellular Pmel17 amyloidogenesis is carefully orchestrated by the secretory pathway, utilizing membrane sequestration and proteolytic steps to protect the cell from amyloid and amyloidogenic intermediates that can be toxic. While functional and pathological amyloid share similar structural features, critical differences in packaging and kinetics of assembly enable the usage of Pmel17 amyloid for normal function. The discovery of native Pmel17 amyloid in mammals provides key insight into the molecular basis of both melanin formation and amyloid pathology, and demonstrates that native amyloid (amyloidin) may be an ancient, evolutionarily conserved protein quaternary structure underpinning diverse pathways contributing to normal cell and tissue physiology.

## Abstract 2

The authors show that native Pmel17 amyloid found in mammalian melanosomes accelerates melanin synthesis.

## Introduction

Proteins typically adopt a well-defined three-dimensional structure, but can misfold and form aggregates with a specific cross-β sheet fold called amyloid [1–4]. The multistep process of amyloidogenesis is linked to a number of diseases, including many resulting in neurodegeneration [5–7]. Nonpathogenic amyloid has not been detected in higher organisms and was unexpected because of the toxicity associated with its formation. We have discovered an abundant mammalian amyloid structure that functions in melanosome biogenesis, challenging the current view that amyloid in mammals is always cytotoxic.

Melanosomes are highly abundant mammalian cellular organelles generated in developmentally specialized cells including melanocytes and retinal pigment epithelium (RPE) [8,9] that reside in the skin and eyes. Melanosome maturation has been demonstrated to require the formation of detergent-insoluble, lumenal Pmel17 fibers [10–12], which are believed to function in polymerization of intermediates in the synthesis of the tyrosine-based polymer melanin [13,14]. Melanin serves as one of nature's chemical defenses against pathogens, toxic small molecules, and UV radiation, and is present in most eukaryotic phyla ranging from fungi to insects and humans [9,15]. The functional requirement for Pmel17 in pigmentation is also well established. In mice, a point mutation in the Pmel17/*silver* locus results in a progressive loss of pigmentation, apparently through loss of melanocyte viability [16–19]. Mutations in Pmel17 orthologs in chicken and zebrafish also result in hypopigmentation [20,21]. Melanosome biogenesis utilizes the secretory and endocytic pathways to direct furin-like, proprotein-convertase-mediated proteolytic processing of the transmembrane glycoprotein Pmel17 [10] in an acidic post-Golgi compartment, yielding a 28-kDa transmembrane fragment (Mβ) and an 80-kDa lumenal fragment (Mα) [12]. Mβ is degraded, whereas Mα self-assembles into fibers that form the core of mature melanosomes [8,10].

Herein we show that fibers in isolated mammalian melanosomes, consisting of Mα, have an amyloid structure. This conclusion is based on the binding of dyes that fluoresce upon interacting with a cross-β sheet structure and on our ability to reconstitute Pmel17 amyloid formation in vitro as demonstrated by a variety of biophysical techniques. The rapidity of recombinant Pmel17 fibrilization is unprecedented, consistent with a process optimized by evolution for function and to avoid the toxicity of pathological amyloidogenesis. Moreover, we have shown that reconstituted Pmel17 amyloid accelerates melanin formation in vitro, apparently by serving as a scaffold that templates the polymerization of highly reactive melanin precursors, probably influencing the resulting structure of melanin as well. Importantly, Mα amyloid may also mitigate the toxicity associated with melanin synthesis by sequestering and minimizing diffusion of highly reactive, toxic melanin precursors out of the melanosome. The utilization of the amyloid fold for a major cellular activity in mammals demonstrates that sequence and folding pathway evolution can harness this ancient structure for physiological purposes.

## Results

**Figure 1:**
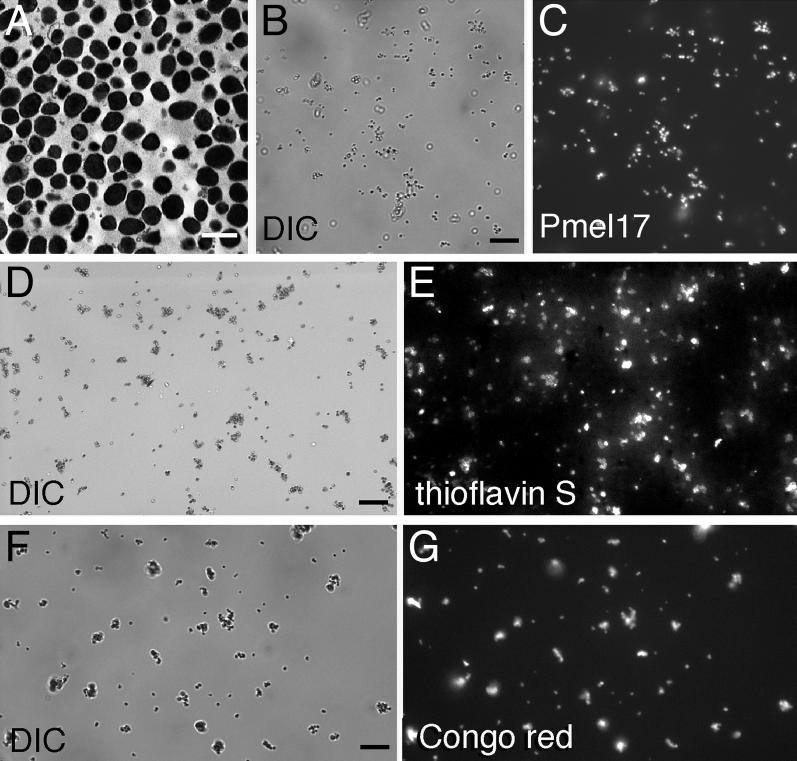
Purified Melanosomes Stain with Amyloidophilic Dyes Melanosomes were isolated from bovine RPE and choroid and visualized using transmission electron microscopy (A; scale bar = 1 μm), differential interference contrast microscopy (DIC) (B, D, and F; scale bars = 10 μm), indirect immunofluorescence using a Pmel17-specific antibody (C), or the thioflavin S (E) or Congo red (G) amyloidophilic fluorophores. Images (B) and (C), (D) and (E), and (F) and (G) are paired.

As a first test to examine whether Mα fibers have an amyloid fold, we prepared a highly enriched melanosome fraction from homogenates of the RPE and choroidal layers from bovine eyes [22] (Figure 1). This ex vivo sampling of melanosomes enables a variety of experiments unavailable in the context of whole tissue while maintaining a high degree of physiological relevance. As expected, purified melanosomes all contained Mα (Figure 1B and 1C). The amyloid content of the melanosomes was interrogated using the amyloid-selective fluorophores thioflavin S and Congo red. These molecules preferentially bind to amyloid over other types of protein aggregates, and their fluorescence upon binding is commonly used in the laboratory and the clinic to diagnose the presence of amyloid in vitro and in vivo [23–25]. Strikingly, greater than 95% of the structures in the melanosome fraction bound thioflavin S and Congo red (Figure 1E and 1G), strongly suggesting that melanosomes contain Mα amyloid. Congo red birefringence was not observed from the stained melanosomes because their thickness is an order of magnitude smaller than the requisite 10-μm thickness achieved in tissue sections. Nevertheless, Congo red fluorescence has been shown to be as effective as birefringence in the diagnosis of amyloid diseases [23].

**Figure 2:**
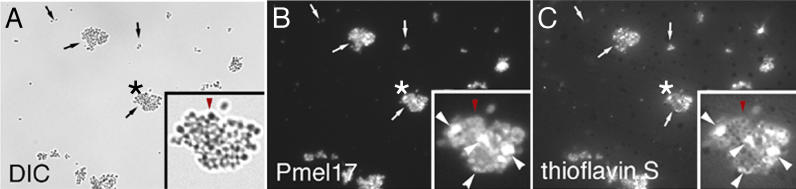
Pmel17 and Thioflavin S Fluorescence Overlap in the Detergent-Insoluble Melanosome Fraction A 1% Triton-X 100 detergent-insoluble fraction was prepared from purified melanosomes and visualized using differential interference contrast microscopy (DIC) (A), indirect immunofluorescence using a Pmel17-specific antibody (B), or thioflavin S fluorescence (C). Arrows denote Pmel17-containing insoluble clusters of variable size. Asterisks indicate the enlarged cluster shown in the lower righthand corner of each panel. In the insets, the large white arrowheads denote Pmel17-positive structures (shown in [B]) that directly overlap with thioflavin S staining (shown in [C]); the red arrowhead in (A) denotes a residual dense melanin-containing granule lacking Pmel17 (shown in [B]) that does not stain with thioflavin S (shown in [C]).

To substantiate that thioflavin S and Congo red binding-induced fluorescence reflect the existence of Mα amyloid, we took advantage of the fact that amyloid fibers resist moderate detergent extraction [5]. When melanosomes were extracted with 1% Triton-X 100 to remove their membranes, thioflavin S–stained particle clusters recovered in the detergent-insoluble fraction showed nearly exclusive overlap with Pmel17 antibody fluorescence (Figure 2), suggesting that Mα is a component of the observed amyloid structures within melanosomes. Thioflavin S did not stain residual melanin-containing dense granules (observed by differential interference contrast microscopy) lacking Mα (Figure 2, red arrowheads in insets), demonstrating that the melanin polymer is not responsible for fluorophore binding in intact melanosomes. Bovine melanosomes lose both Mα and thioflavin S binding upon boiling with 10% sodium dodecyl sulfate (data not shown), consistent with the denaturation of the Mα amyloid fiber under these conditions. Because boiling in 10% sodium dodecyl sulfate does not alter the covalent structure of melanin, these results provide further evidence that thioflavin S is specific for the Mα amyloid component of melanosome granules.

**Figure 3:**
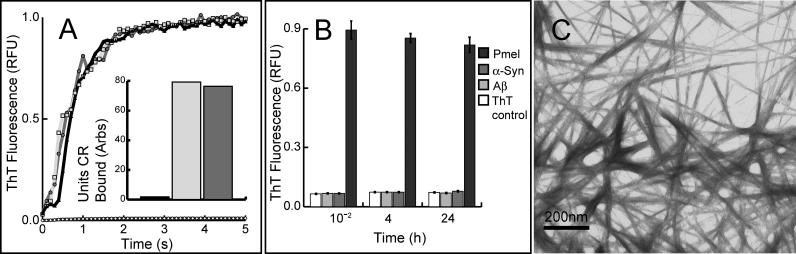
rMα Rapidly Forms Thioflavin T– and Congo Red–Positive Fibers under Nondenaturing Conditions (A) rMα samples (in 8 M GdmCl to preserve an unfolded, nonaggregated state) were diluted by manual mixing to start an amyloid fiber formation time course (monitored by thioflavin T fluorescence) at varying pHs: pH 7.4 (black line, triangles), pH 6.0 (dark grey line, circles), and pH 4.85 (light grey line, squares); control (thioflavin T buffer) (black line, white diamonds ). The inset bar graph reflects endpoint Congo red binding of equimolar amounts of deposits of Mα formed at pH 7.4 (dark grey), Aβ 1–40 fibers associated with Alzheimer disease (light grey), and control (Congo red buffer) (black). (B) rMα (Pmel) forms thioflavin T (ThT)–positive aggregates at least four orders of magnitude faster than either α-synuclein (α-Syn) or Aβ when all three polypeptides are diluted from 8 M GdmCl into physiological buffer (error bars represent the standard deviation of triplicate samples). (C) Transmission electron micrograph of typical rMα amyloid fibers with an average diameter of 10 nm, formed under nondenaturing conditions.

The data suggest that the Mα fibers found in melanosomes within mammalian cells have an amyloid fold. Consistent with this, we found that Mα spontaneously self-assembles into amyloid in vitro. The non-glycosylated, 442-residue lumenal fragment of Pmel17, referred to as recombinant Mα (rMα; without carbohydrate) was reconstituted from *Escherichia coli* inclusion bodies. Purification of rMα by gel filtration in 8 M guanidinium hydrochloride (GdmCl) was required to preserve an unfolded, nonaggregated state (Figure S1). Dilution of rMα into nondenaturing buffers resulted in exceedingly rapid amyloidogenesis (within 3 s, rate-limited by the time required for mixing) over a broad pH range as monitored by thioflavin T fluorescence (Figure 3A) and endpoint Congo red analysis (Figure 3A, inset). To ensure that rMα amyloidogenesis kinetics were not altered by the presence of GdmCl-resistant Mα seeds, stock rMα solutions purified by gel filtration in 8 M GdmCl were subjected to ultracentrifugation (500,000 *g* for 1 h) before the top 90% of supernatant was removed and subjected to chaotrope-dilution-initiated amyloidogenesis. Ultracentrifugation did not decrease the rate of amyloidogenesis or alter the concentration of the rMα stock. Other highly amyloidogenic, natively unfolded proteins such as Aβ and α-synuclein do not form amyloid within the dead time of mixing upon transfer from denaturing to nondenaturing conditions (Figure 3B). In fact, rMα amyloidogenesis is at least four orders of magnitude faster than that of Aβ or α-synuclein under identical physiological conditions at room temperature (Figure 3A and 3B). We are unaware of any other protein nearly so amyloidogenic [26], consistent with a functional amyloid fold optimized by evolution to avoid the formation of toxic intermediates prominent in pathogenic amyloidogenesis [7].

**Figure 4:**
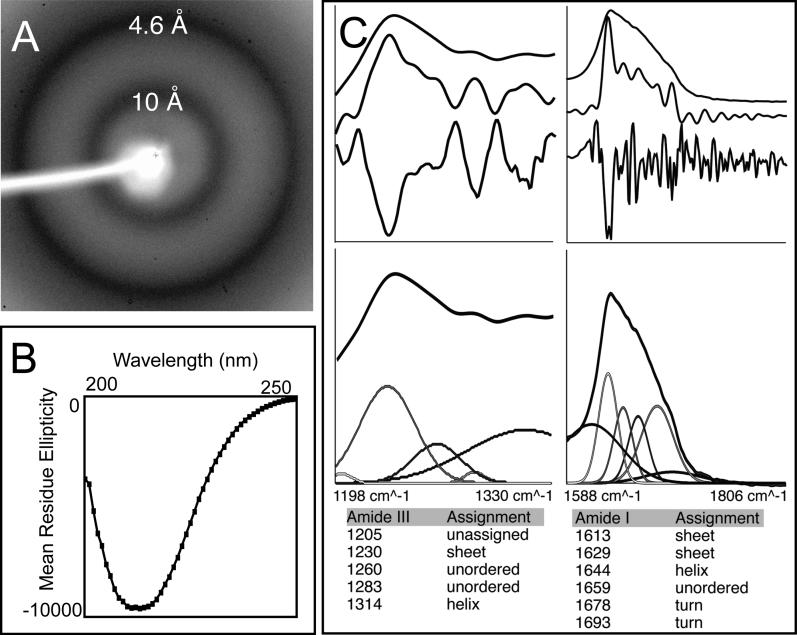
rMα Fibers Have a Cross-β Sheet Structure (A) X-ray powder diffraction of lyophilized rMα fibers formed in vitro exhibit a very strong reflection at 4.6 Å and a strong reflection at 10 Å, which is expected of an amyloid cross-β sheet structure. (B) The far-UV CD spectra of soluble Mα aggregates formed at low concentrations to avoid precipitation support a predominantly β-sheet structure. Mα aggregates are approximately 11% α-helix, 32% β-sheet, 23% β-turn, and 33% disordered, based on curve fitting with a basis set of 43 soluble proteins. Since β-sheet content is estimated using a set of proteins not composed of cross-β sheet structures, the potential error in the estimate cannot be determined. (C) The attenuated total reflectance FT-IR spectrum of aggregated rMα in the solid state supports a β-sheet-rich structure. Peaks in the amide III (top left, upper curve) and I (top right, upper curve) regions were identified using Fourier self-deconvolution (top left and right, middle curve) and confirmed by second derivative analysis (top left and right, bottom curve). Peak assignments are listed, and were used to fit the original spectrum using fixed Gaussian peaks at the assigned positions (bottom). Peaks assigned to β-sheet regions of the spectrum accounted for a large percentage of the total area in the amide I and III regions.

A variety of structural techniques were employed to confirm that rMα aggregates possess an amyloid structure. Aggregate morphology was examined by electron microscopy, revealing fibers with an average diameter of 10 nm, typical of amyloid formed in vitro (Figure 3C) [27]. X-ray powder diffraction of rMα aggregates revealed a very strong reflection at 4.6 Å and a weaker reflection at 10 Å (Figure 4A), consistent with the amyloid cross-β sheet quaternary structure [28]. The presence of β-sheet structure is further supported by the far-UV circular dichroism (CD) spectrum of soluble rMα amyloid formed at low concentrations (Figure 4B) and the attenuated total reflectance Fourier transform infrared (FT-IR) spectrum of insoluble rMα amyloid, which are both fully consistent with known amyloid spectra (Figure 4C) [29]. Both methods indicate approximately 50% β-sheet content, suggesting that rMα amyloid may contain folded domains external to the fiber structure. The Ure2p prion amyloid is known to have a central amyloid core with an attached globular domain [30]. Our biophysical data revealed that the Mα fibers required for melanosome biogenesis [10,12] are amyloid.

**Figure 5:**
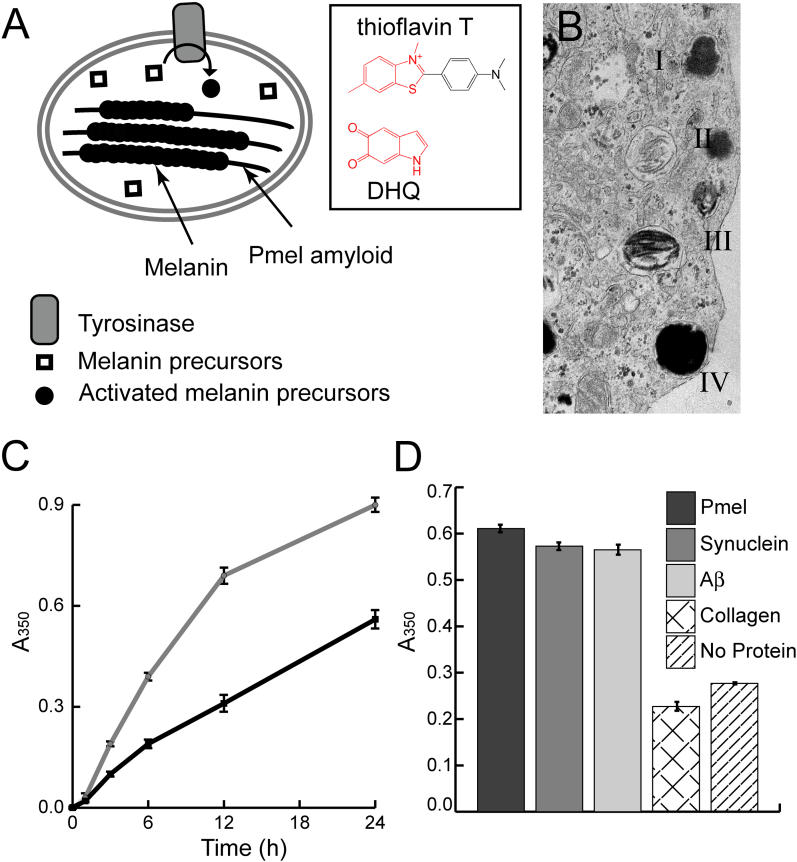
Amyloid, Including rMα, Specifically Accelerates Melanin Synthesis (A) In melanosomes, assembly of activated melanin precursors, generated by tyrosinase, occurs along Pmel17 fibers. The boxed portion of (A) illustrates the amyloid-binding dye thioflavin T and the activated melanin precursor DHQ, which possess similar core structures. This suggests an explanation for the ability of Pmel17 to concentrate and organize melanin precursors, thereby enabling melanogenesis. (B) In vivo, melanosome maturation is a four-step process (I–IV) in which initial formation of the Pmel17 fibrillar matrix (II) enables subsequent melanin polymerization along the Pmel17 fibers (III) (Adapted with permission from [16].) (C) A time course of melanin synthesis in vitro shows that insoluble rMα amyloid increases the amount of insoluble melanin formed per unit time (grey line) versus a control reaction lacking rMα (black line). (D) Melanin synthesis after 20 h was also evaluated in the presence of insoluble rMα amyloid, α-synuclein amyloid, Aβ amyloid, and collagen IV α-helical fibers. The melanin precursor D,L-DOPA was incubated in the presence of the enzyme tyrosinase and the amyloid of interest at room temperature. Melanin content of each reaction condition was measured by pelleting insoluble melanin, dissolving it in 1 M NaOH, and measuring the absorbance at 350 nm. Supernatant melanin content was equal for all samples. In (C) and (D) error bars represent the standard deviation between triplicate samples.

rMα amyloid was used to further investigate the native function of Mα amyloid fibers in melanogenesis. The monomeric precursor for melanin polymerization, indole-5,6-quinone (DHQ), is one of the terminal products of a series of oxidation steps initiated by the action of the type I transmembrane enzyme tyrosinase on the substrate tyrosine [15]. Melanin is thought to consist of DHQ and other intermediates polymerized upon a template of Mα fibers within the maturing melanosome (Figure 5A and 5B). To test this possibility, we employed an in vitro assay utilizing tyrosinase, 3,4-dihydroxyphenylalanine (DOPA), and rMα amyloid that recapitulates melanin formation within the melanosome (Figure 5C and 5D). A time course reveals that rMα amyloid hastens the formation of insoluble melanin when added to the melanization assay (Figure 5C), resulting in more melanin per unit time. rMα amyloid also affords more than 2.2-fold more melanin after 20 h than an equivalent amount of collagen IV, an α-helical fiber (Figure 5D). Interestingly, DHQ shares a core that is isostructural with the benzothiazole substructure of the amyloidophilic fluorophore thioflavin T (Figure 5A, box), which might account for its affinity for amyloid fibers. Recent studies have shown that thioflavin T binds in a regular fashion parallel to the amyloid fiber axis [31]. Binding of DHQ in an analogous fashion may be what enables Mα amyloid to concentrate and organize reactive DHQ or analogous reactive melanin precursors along the Mα fiber, templating their efficient covalent polymerization. Because the Mα fibers are completely buried during the process of melanogenesis they are unlikely to function as catalysts. Strikingly, α-synuclein and Aβ amyloid enhance the yield of melanin formation in a manner comparable to rMα amyloid in our in vitro melanogenesis assay (Figure 5D). Apparently, the cross-β sheet structure of amyloid, shared between Mα, Aβ, and α-synuclein fibers, functions specifically to template the synthesis of melanin in vitro. We suggest that this process occurs in vivo on Mα fibers within melanosomes.

## Discussion

The discovery of amyloid as a prominent structure in eukaryotic cells now adds the amyloid fold to the repertoire of structures used in normal mammalian cell physiology. We provide a number of lines of in vitro and ex vivo evidence to support this conclusion. Ex vivo melanosomes exhibit selective environment-sensitive thioflavin T and Congo red fluorescence and have detergent-resistant properties expected of amyloid. rMα assembles faster than any known polypeptide into amyloid fibers, at least four orders of magnitude faster than the Aβ and α-synuclein peptides associated with Alzheimer and Parkinson disease, consistent with an evolved sequence that adopts an amyloid structure. The structure produced spontaneously by Mα in vitro exhibits the characteristic X-ray fiber diffraction, CD and FT-IR spectra, and fibrillar morphology expected of amyloid. Finally, rMα amyloid fibers can hasten melanin formation in vitro by serving as a template for DHQ polymerization, thereby recapitulating the fibers' putative native function [16].

Given the toxic nature of amyloid and its precursors in both intracellular and extracellular contexts, it would be expected that Mα fibrillogenesis be highly regulated to avoid damage to the cell. Indeed, full-length Pmel17 is synthesized and trafficked to early melanosomes as a transmembrane protein incapable of self-assembly. Only when sequestered in the specialized early melanosome compartment is the amyloidogenic fragment, Mα, released by proteolysis. The rapid self-assembly of Mα in combination with its membrane sequestration presumably minimizes the toxicity usually associated with amyloidogenesis. Other proteins may also be involved in the initiation and regulation of Mα amyloidogenesis [32], as is the case for functional *E. coli* and *Salmonella* extracellular curli fibers [33], extracellular spider silk fibers [34], Sup35 amyloid in yeast [35], and, potentially, CPEB prions [36].

While functional amyloidogenesis exhibits some similarities to pathogenic amyloid formation, it also displays striking differences. In gelsolin amyloid disease, proteolysis of mutant gelsolin during secretion by the proprotein convertase furin leads to slow, unregulated extracellular pathogenic gelsolin amyloid deposition [37]. Mα amyloid formation is also initiated by proprotein convertase activity, but the product is a functional amyloid structure confined to a membrane-delimited compartment. The rapidity of rMα amyloidogenesis is likely important, as this may preclude the formation of toxic, diffusible intermediates that could compromise cellular integrity [5,7,11]. These key differences in packaging and assembly appear to enable the usage of amyloid as a major intracellular structure for normal function. Study of functional Mα amyloid is likely to provide critical insights into the pathological basis for important misfolding diseases including Huntington, Parkinson, and Alzheimer disease, where the biological contexts and folding constraints differentiating normal from pathological folds are currently not appreciated.

Mα fibers in melanosomes serve to bind and orient highly reactive melanogenic precursors, hastening their polymerization and likely influencing the resulting melanin structure. Another apparently important function of Mα amyloid is to prevent cytotoxicity associated with the process of melanin polymerization, and hence melanosome biogenesis. Highly reactive, uncharged hydrophobic melanin precursor compounds would be expected to diffuse across the membrane and enter the cytoplasm if they were not sequestered by Mα fibers, upon which they polymerize. Large excesses of melanogenic precursors have been shown to produce severe cytotoxic effects in melanizing cells [38], and Pmel17 mutations leading to minimal Mα amyloid fiber formation result in reduced melanocyte viability [16–19]. These observations can be explained by the leakage of toxic melanogenic intermediates from the melanosome as a result of insufficient sequestration by Mα amyloid. Hence, the ability of Mα fibers to bind and concentrate these reactive precursors appears to protect the cell against the toxicity that can result from melanosome biogenesis.

The discovery that a major mammalian biosynthetic pathway utilizes a cross-β sheet structure for function establishes the amyloid fold as a key protein structural motif utilized by a wide variety of organisms from prokaryotes to humans. Melanin polymer chemistry plays a wide variety of roles in an array of organisms—it is involved in pigmentation and cytoprotection in higher eukaryotes, it is critical for stress responses and structural stability in plants, and it is an integral component of the insect immune system [15]. Mα amyloid has a critical role in melanin formation in humans, and is the first example to our knowledge of an amyloid that functions in a chemical reaction, pointing the way towards the discovery of amyloid in other important functional roles. It is now apparent that the amyloid fold has been selected multiple times during evolution for a variety of functions. Given the propensity of most polypeptides to form amyloid in vitro [5], the usage of amyloid in biology may be as common as other canonical protein folds. This contrasts with the current view that there is evolutionary pressure against amyloidogenesis. We suggest that the amyloid fold is a fundamental protein structural motif with unique properties that is capable of performing a wide variety of functions. We propose the general name *amyloidin* for functional amyloid, with the expectation that the number and diversity of structures of this type will continue to grow.

## Materials and Methods

### Immunofluorescence

Bovine melanosomes were fixed in methanol at −20 °C and blocked with 5% BSA/2% normal goat serum in TBS for 10 min at room temperature. Melanosomes were incubated with a chicken polyclonal anti-Pmel17 antibody, GP100 (Zymed, San Francisco, California, United States), for 1 h at room temperature at a dilution of 1:150. The secondary antibody (goat anti-chicken IgG rhodamine, Molecular Probes, Eugene, Oregon, United States) was used at a dilution of 1:200 for 1 h at room temperature. Melanosomes were then washed and mounted with PBS and imaged.

### Staining with thioflavin S and Congo red

Thioflavin S and Congo red staining were carried out as previously described [25]. Briefly, for thioflavin S, purified bovine melanosomes were thawed, washed once with PBS, and stained for 1 h in a 1% (w/v) solution of thioflavin S in water. Melanosomes were then washed twice with 80% ethanol and once with PBS. For Congo red, melanosomes were thawed, washed once with PBS, and stained using the alkaline Congo red method [25]. Melanosomes were then washed twice with absolute ethanol. Detection of amyloidin encapsulated in granules requires incubation for longer than typical times reported for extracellular pathogenic amyloid.

### Melanosome purification and extraction

Melanosomes were isolated from the RPE and choroid layers of bovine eyes by sucrose density ultracentrifugation as previously described [22] with minor modifications. The RPE cell layer was collected in 0.25 M sucrose buffer (10 mM Tris [pH 7.4], 65 mM NaCl, 2 mM MgCl_2_, protease inhibitor cocktail [Sigma, St. Louis, Missouri, United States]) and disrupted by Dounce homogenization. The homogenate was centrifuged at 2,000 *g* at 4 °C for 10 min to obtain a postnuclear supernatant. The postnuclear supernatant was layered onto a sucrose step gradient (0.75 M/1.5 M/2 M sucrose) and centrifuged at 85,000 *g* for 1 h. The melanosome-rich fraction was collected from the 2 M layer of the gradient and washed in 0.25 M sucrose buffer. To prepare the detergent-insoluble fraction, isolated melanosomes were resuspended in extraction buffer (150 mM NaCl, 100 mM Tris, 0.1% NaN_3_). Triton-X 100 was added from a 10% stock solution to a final concentration of 1%. The suspension was shaken at 4 °C for 2 h. The insoluble fraction was collected by centrifugation at 10,000 *g* for 1 min and washed three times with extraction buffer.

### rMα expression and purification

The lumenal fragment of Pmel17, rMα, consisting of amino acids 25–467 was subcloned into a pET3c vector and expressed in BL21-DE3 *E. coli*. Shaken cultures (1 l) were grown at 37 °C to OD_600_ = 0.5 in the presence of 270 μM ampicillin and then induced with 1 μM IPTG for 4 h. Cells were collected via centrifugation at 4 °C, resuspended in TBS, and frozen at −80 °C. The resuspended pellet was thawed and the cells were lysed by probe sonication. rMα formed inclusion bodies that were collected by centrifugation, and washed by resuspension followed by centrifugation twice in washing buffer (1.5 M NaCl, 50 mM KH_2_PO_4_/K_2_HPO_4_ [pH 7.4], 1% Triton-X 100) and then in TBS. The inclusion body pellet was dissolved in extraction buffer (8 M GdmCl, 50 mM KH_2_PO_4_/K_2_HPO_4_ [pH 7.4], 100 mM KCl, 5 mM EDTA) by magnetic stirring at 4 °C for 48 h. The resulting solution was centrifuged, filtered through a 0.22 μM cellulose acetate filter, and then frozen at −80 °C. After thawing, the solution was gel filtered using extraction buffer with a Superdex 200 26/60 column. Purified rMα fractions were assayed via SDS-PAGE and Western blot using the GP100 anti-Pmel17 antibody. rMα was concentrated using a 3-kDa MWCO Centricon filter (Millipore, Bedford, Massachusetts United States) and stored at room temperature.

### Thioflavin T binding assays

Thioflavin T binding kinetics were assayed using a Cary Eclipse fluorimeter (Varian, Palo Alto, California, United States). Assay buffer (50 mM KH_2_PO_4_/K_2_HPO_4_, 100 mM KCl, 5 mM EDTA, 20 μM thioflavin T) at the appropriate pH was placed in a stirred cuvette. The solution was excited at 440 nm and data were collected at 485 nm. After data collection had been initiated, an aliquot of concentrated rMα in extraction buffer was rapidly added to a final concentration of 10 μM. Data shown represent several normalized traces that have been averaged. Stagnant thioflavin T binding kinetics were assayed using an Aviv (Lakewood, New Jersey, United States) ATF105 fluorimeter. Stock solutions of Aβ, α-synuclein, and rMα in 8 M GdmCl, 50 mM KH_2_PO_4_/K_2_HPO_4_ (pH 7.4), and 100 mM KCl were diluted to a final concentration of 10 μM in assay buffer at pH 7.4. The solutions were allowed to aggregate for various amounts of time, whereupon thioflavin T was added from a stock solution to a final concentration of 20 μM. The samples were excited at 440 nm and data were collected at 485 nm. Control experiments were performed to ensure that guanidine-resistant seeds were not affecting Mα amyloidogenesis rates using stock rMα solutions in 8 M GdmCl that were centrifuged for 1 h at 500,000 *g*. These controls gave identical time courses to experiments in which the centrifugation step was not performed.

### Congo red binding assay

An aliquot of concentrated rMα was added to assay buffer (50 mM KH_2_PO_4_/K_2_HPO_4_, 100 mM KCl, 5 mM EDTA). The solution was vortexed and allowed to incubate for 5 min, whereupon Congo red was added from a concentrated stock solution to a final concentration of 20 μM. Absorbance spectra were recorded using a Hewlett-Packard (Palo Alto, California, United States) 8453 spectrometer. Units of Congo red bound were calculated using the following equation: OD_540_/25,295 − OD_477_/46,306 [39].

### Fluorescence microscopy

Images were captured using a Zeiss (Oberkochen, Germany) Axiophot epi-fluorescence microscope attached to an Axiocam digital camera using the following filter configurations: Pmel17 antibody (excitation 545 nm, emission 560–625 nm), thioflavin S (excitation 436 nm, emission > 455 nm), and Congo red (excitation 530–585 nm, emission > 600 nm).

### CD secondary structure analysis

CD spectra were collected on an Aviv 202A CD spectrometer. Concentrated rMα in extraction buffer was diluted into filtered, de-ionized water to a final protein concentration of 3 μM (336 mM GdmCl, 2.1 μM KH_2_PO_4_/K_2_HPO_4_, 4.2 μM KCl, 0.21 μM EDTA). Ten spectra were averaged and background corrected before analysis of secondary structure components using averages of the outputs of Cdsstr, SELCON, and CONTIN algorithms with a basis set of 43 soluble proteins [40–42].

### FT-IR secondary structure analysis

Concentrated rMα in extraction buffer was dialyzed versus filtered, deionized water. Approximately 100 μL of this 50 μM rMα aggregate solution was deposited on a Ge-attenuated total reflectance infrared cell and allowed to dry under flowing nitrogen. Three thousand scans were acquired on a Nicolet Magna 550 FT-IR (Thermo Electron, Madison, Wisconsin, United States) using dried dialysis buffer as a background. The spectra were smoothed using a nine-point Savitsky-Golan algorithm, and peaks were identified using Fourier self-deconvolution as well as a second-derivative analysis. Assignments in the amide I and amide III regions were made based on literature precedent [29,43]. Spectral sections corresponding to the amide I (1,588–1,806 cm^−1^) and amide III (1,198–1,330 cm^−1^) regions were fit using Gaussian peaks with fixed positions. Gross estimates of secondary structure were made based on the relative size of the various peaks.

### X-Ray powder diffraction

Concentrated rMα in extraction buffer was dialyzed versus MilliQ water. Aggregates were lyophilized, producing a fine powder. Powder diffraction of approximately 1.0 mg of rMα in quartz capillaries was recorded using a 6-kW Bruker (Madison, Wisconsin, United States) Direct Drive Rotating anode X-ray generator with a Xenocs (Sassenage, France) focusing mirror (50 kV × 100 mA, 0.3 × 3 mm focus, 0.5 mm slits, copper target) and a Mar 345-mm IP scanner. The distance from sample to scanner was 250 mm and CuK_α_ radiation (1.5418 Å) was utilized.

### Electron microscopy of rMα fibers

rMα fibers were generated by diluting (from concentrated 8 M GdmCl, 50 mM KH_2_PO_4_/K_2_HPO_4_ [pH 7.4], 100 mM KCl stock) rMα into 125 mM CH_3_COOH/CH_3_COOK buffer (pH 5.0) at a final concentration of 10 μM and allowing it to stand at room temperature for 24 h. rMα aggregates were adsorbed onto 200-mesh carbon-coated copper grids (Electron Microscopy Sciences, Hatfield, Pennsylvania, United States), stained with 1% aqueous uranyl acetate (Electron Microscopy Sciences), and visualized with a Philips (New York, New York, United States) CM100 transmission electron microscope.

### Synthetic melanogenesis

The ability of rMα to enhance melanogenesis was evaluated using D,L-DOPA (Sigma) and tyrosinase (Calzyme, San Louis Obispo, California, United States). rMα (0.5 mg) was added to 1.0 ml of fresh assay buffer (5.0 mM D,L-DOPA, 125 mM CH_3_COOH/CH_3_COOK buffer [pH 5.0]) from a concentrated stock solution (8 M GdmCl, 50 mM KH_2_PO_4_/K_2_HPO_4_ [ph 7.4], 100 mM KCl). Aβ was purchased (SynPep, Dublin, California, United States) and rendered seed-free by dissolving in water, sonicating for 15 min, adjusting the pH to 10.5 using 100 mM NaOH, sonicating for 15 min, filtering through a 0.22-μm syringe filter, and finally filtering through a 10-kDa MWCO concentrator (Centricon). The resulting solution (400 μM Aβ) was mixed in equal volumes with a solution of 600 mM NaCl, 300 mM NaPO_4_ (pH 7.5), and 0.04% NaN_3_ and allowed to form amyloid with rocking at 37 °C for 4 d. α-Synuclein was expressed and purified from *E. coli* using a procedure adapted from Lashuel et al. [44]. Cultures were grown to OD_600_ = 0.5 and then induced for 12 h with 1 mM IPTG. Cells were harvested and lysed using probe sonication at 4 °C, and the supernatant collected. Streptomycin sulfate (1% [w/v]) was added to the supernatant, which was then stirred at 4 °C for 60 min. The precipitated proten was removed by centrifugation, NH_4_SO_4_ (0.129 g/ml) was added, and the solution was stirred at 4 °C for 60 min. The supernatant was decanted and the pellet resuspended in one-tenth of the culture volume of 10 mM Tris buffer (pH 7.4). The protein was then purified on a source Q column (buffer A, 10 mM Tris [pH 7.4]; buffer B, A + 1 M NaCl). α-Synuclein-containing fractions were concentrated to one-tenth of their original volume and further purified using a Superdex 200 26/60 column with 100 mM (NH_4_)_2_CO_3_. The purified α-synuclein was then lyophilized and stored at −80 °C until used. Lyophilized α-synuclein was dissolved in 25 mM MES buffer (pH 6.0) and induced to form amyloid by rocking at 37 °C for 48 h. The amyloid nature of the α-synuclein and Aβ aggregates was tested by far-UV CD (which showed β-sheet) and TEM or AFM (which showed fibers). α-Synuclein or Aβ amyloid was collected by centrifugation (16,000 *g* for 15 min) and resuspended in assay buffer at a final concentration of 0.5 mg/ml. Collagen IV (BD Biosciences, Franklin Lakes, New Jersey, United States) was rendered insoluble by lyophilization and resuspended in assay buffer at a final concentration of 0.5 mg/ml. These solutions were vortexed, and 10 μg of tyrosinase was added. The solutions were allowed to react at room temperature for varying periods of time, after which they were centrifuged at 16,000 *g* for 15 min. The pellets (insoluble rMα, α-synuclein, collagen IV, and melanin products) were resuspended in 125 mM CH_3_COOH/CH_3_COOK buffer (pH 5.0) and centrifuged again. The pellets were then resuspended in 1 M NaOH and then heated to 60 °C for 5 min and vortexed to effect dissolution. Absorbance spectra were recorded at 350 nm.

## Supporting Information

Figure S1 The Intrinsic Tryptophan Fluorescence (Excitation 295 nm) of rMα Was Measured in 8 M GdmCl and in Nondenaturing Buffer rMα tryptophan emission in nondenaturing buffer is significantly blue-shifted with respect to rMα tryptophan emission in 8 M GdmCl, most likely owing to aggregation-induced burial and shielding of the tryptophan residues from the aqueous buffer. The red-shifted data indicate that rMα is unfolded in 8 M GdmCl, consistent with observations using gel filtration chromatography.
(102 KB TIF).

### Accession Numbers

The Swiss-Prot (http://www.ebi.ac.uk/swissprot/) accession numbers for the gene products discussed in this paper are Pmel17 (P40967) and type I transmembrane enzyme tyrosinase (P14679).

## Abbreviations

CD: circular dichroism
FT-IR: Fourier transform infrared
DHQ: indole-5,6-quinone
DOPA: 3,4-dihydroxyphenylalanine
GdmCl: guanidinium chloride
rMα: recombinant Mα
RPE: retinal pigment epithelium
